# Subcellular Distribution of Glutathione Precursors in *Arabidopsis thaliana*

**DOI:** 10.1111/j.1744-7909.2011.01085.x

**Published:** 2011-12-13

**Authors:** Barbara Eva Koffler, Romana Maier, Bernd Zechmann

**Affiliations:** 1 University of Graz, Institute of Plant Sciences8010 Graz, Austria; 2 Graz University of Technology, Institute for Electron Microscopy and Fine Structure Research8010 Graz, Austria

**Keywords:** *Arabidopsis*, cysteine, glutamate, glutathione, glycine

## Abstract

Glutathione is an important antioxidant and has many important functions in plant development, growth and defense. Glutathione synthesis and degradation is highly compartment-specific and relies on the subcellular availability of its precursors, cysteine, glutamate, glycine and γ-glutamylcysteine especially in plastids and the cytosol which are considered as the main centers for glutathione synthesis. The availability of glutathione precursors within these cell compartments is therefore of great importance for successful plant development and defense. The aim of this study was to investigate the compartment-specific importance of glutathione precursors in *Arabidopsis thaliana*. The subcellular distribution was compared between wild type plants (Col-0), plants with impaired glutathione synthesis (glutathione deficient *pad2-1* mutant, wild type plants treated with buthionine sulfoximine), and one complemented line (OE3) with restored glutathione synthesis. Immunocytohistochemistry revealed that the inhibition of glutathione synthesis induced the accumulation of the glutathione precursors cysteine, glutamate and glycine in most cell compartments including plastids and the cytosol. A strong decrease could be observed in γ-glutamylcysteine (γ-EC) contents in these cell compartments. These experiments demonstrated that the inhibition of γ-glutamylcysteine synthetase (GSH1) – the first enzyme of glutathione synthesis – causes a reduction of γ-EC levels and an accumulation of all other glutathione precursors within the cells.

## Introduction

The tripeptide glutathione (γ-glutamylcysteinylglycine) is an important antioxidant in plants and is of great importance for plant metabolism and plant defense. It plays important roles in the detoxification of reactive oxygen species (Noctor and Foyer 1998; Tausz et al. 2004; Foyer and Noctor 2009; Szalai et al. 2009; Noctor et al. 2011), xenobiotics, herbicides (Edwards et al. 2005; DeRidder and Goldsbrough 2006), heavy metals such as cadmium (Zawoznik et al. 2007; Ammar et al. 2008; DalCorso et al. 2008; Dučić et al. 2008; Nocito et al. 2008), and protects proteins from oxidation through a process called glutathionylation (Hurd et al. 2005a, 2005b). Glutathione is also involved in stress signaling and defense gene expression (Foyer et al. 2001; Maughan and Foyer 2006; Foyer and Noctor 2009; Szalai et al. 2009; Noctor et al. 2011). Considering the importance of glutathione, its availability, synthesis, and degradation in plants is of great importance for successful plant development and defense.

Glutathione synthesis underlies, like its degradation, highly compartment-specific pathways (Cairns et al. 2006; Pasternak et al. 2008; Noctor et al. 2011). Glutathione synthesis in plants takes place in two adenosine triphosphate (ATP)-dependent steps. In the first step, cysteine is linked together with glutamate by γ-glutamylcysteine synthetase (GSH1; also referred to as γ-ECS in some literature) to form γ-glutamylcysteine (γ-EC). As GSH1 is encoded by a single gene, which is exclusively targeted to plastids in *Arabidopsis,* it is speculated that this reaction takes place exclusively in plastids in *Arabidopsis* plants (Wachter et al. 2005). The situation is less clear in other plant species as GSH1 has also been detected in leaf extracts (e.g. wheat) after chloroplast isolation (Noctor et al. 2002) and as it is encoded by more than one gene in some plant species (e.g. *Oryza sativa*, *Populus trichocarpa*). Thus, it appears that in other plant species GSH1 might also be active in cell compartments (e.g. cytosol) other than the chloroplast (Hell and Bergmann 1990; Noctor et al. 1998; Noctor et al. 2002; Kopriva 2006). In the second, step glycine is linked to γ-EC by glutathione synthetase (GSH2; also referred to as GSHS in some literature) to form the final product, glutathione. As GSH2 is targeted to plastids and the cytosol in *Arabidopsis* (Wachter et al. 2005) it seems that this step takes place, to different extents, in both plastids and the cytosol (Noctor et al. 2002; Sugiyama et al. 2004). Thus, considering the current knowledge about glutathione synthesis, plastids and the cytosol can be considered the main centers of glutathione synthesis in plants (Noctor et al. 2011). Cysteine and subsequently γ-EC are the limiting precursors for glutathione synthesis as it has been demonstrated that both the artificial elevation of cysteine (Gullner et al. 1999; Harms et al. 2000; Bloem et al. 2004; Bloem et al. 2007; Zechmann et al. 2007a, 2008a) and the overexpression of genes and enzymes involved in cysteine synthesis (Harms et al. 2000; Noji and Saito 2002; Wirtz and Hell 2007) increased glutathione in plants. Nevertheless, it has been shown, under certain conditions (absence of photorespiration, darkness), that glycine can also limit glutathione synthesis (Noctor et al. 1997a, 1997b). Further, since the first step of glutathione synthesis in *Arabidopsis* seems to take place exclusively in plastids, the availability of cysteine and glutamate in plastids is essential for the synthesis of γ-EC and subsequently glutathione. In addition, as the second step of glutathione synthesis takes place in plastids and the cytosol, the availability of glycine and γ-EC determines glutathione synthesis in these two cell compartments. Thus, it becomes obvious that the subcellular determination of glutathione precursors and the correlation to the compartment-specific glutathione status is essential for the better understanding of glutathione synthesis and its role in plant development and defense. Despite our deep understanding of the compartmentation of glutathione synthesis, the compartmentation of glutathione degradation is still strongly debated. Most attention continues to be paid to apoplastic and vacuolar routes, but another possibility of glutathione degradation also involves the cytosol (Noctor et al. 2011). One possible pathway of glutathione degradation is catalysed by γ-glutamyl transpeptidase (also referred to as γ-glutamyl transferase in some literature), (GGT) which transfers glutamate from glutathione to other dipeptides. Isoforms of GGT occur at the plasmalemma, within vacuoles and the apoplast (Foyer et al. 2001; Noctor et al. 2001; Storozhenko et al. 2002; Shaw et al. 2005; Ohkama-Ohtsu et al. 2007a, 2007b; Ferretti et al. 2009; Destro et al. 2011). Another pathway of glutathione degradation is facilitated by a carboxypeptidase that has been detected within vacuoles of barley leaves (Wolf et al. 1996) and removed glycine from glutathione. The remaining dipeptides in both pathways could then be metabolized by a dipeptidase to the component amino acids (Foyer et al. 2001). Other pathways of glutathione degradation could be facilitated by γ-glutamyl cyclotransferase and 5-oxoproline, which produce free glutamate (Martin and Slovin 2000; Ohkamu-Ohtsu et al. 2008), or by phytochelatin synthase, which could be especially important for the breakdown of glutathione in situations where conjugated glutathione accumulates within the cytoplasm (Grzam et al. 2006; Blum et al. 2007, 2010). Both reactions seem to take place in the cytosol and would be alternative pathways to the degradation of glutathione in the vacuole and the apoplast (Noctor et al. 2011).

The aim of this study was to investigate the subcellular distribution of glutathione precursors during situations of impaired glutathione synthesis in order to obtain more information about the compartment-specific importance of glutathione precursors for glutathione synthesis and degradation. The hypothesis that low levels of glutathione induced by the inhibition of GSH1 are caused by low levels of γ-EC rather than by reduced production of the other glutathione precursors was tested. Therefore the subcellular distribution of cysteine, glutamate, glycine and γ-EC was studied and correlated with the compartment-specific distribution of glutathione in the *Arabidopsis* mutant *pad2-1* and one complemented line (OE3, which is equivalent to line 3 described in Parisy et al. 2007) where glutathione synthesis was restored by genetic complementation of *pad2-1* with wild-type GSH1 cDNA (Parisy et al. 2007; Zechmann et al. 2008b). The *pad2-1* mutant shows impaired glutathione metabolism due to a single point mutation of the gene encoding GSH1 thus leading to about 80% less glutathione contents when compared with wild type (Parisy et al. 2007; Zechmann et al. 2008b). The situation in the *pad-2-1* mutant was compared with plants where glutathione synthesis was artificially inhibited by buthionine sulfoximine (BSO) for 2 days in order to investigate the effects of long and short term inhibition of glutathione synthesis on glutathione precursor contents. BSO inhibits GSH1, thus leading to a strong decrease in glutathione contents in plants (Müller et al. 1999; Gullner and Dodge 2000; Meyer and Fricker 2002; Hartmann et al. 2004; Senda and Ogawa 2004; Zechmann et al. 2006b). During BSO-treatment the investigations of the subcellular distribution of glutathione precursors in the above described mutants gave more detailed insight into the compartment-specific importance of glutathione precursors during situations of impaired glutathione production and for glutathione synthesis and degradation in general.

## Results

### Cysteine

**Figure 1:**
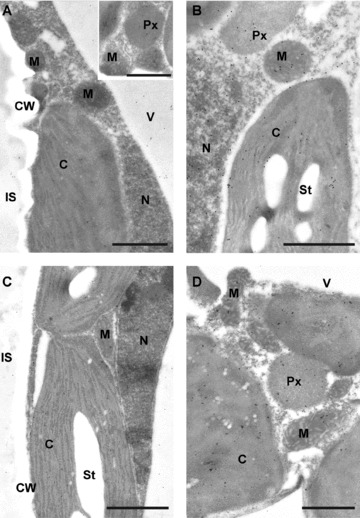
**Transmission electron micrographs of mesophyll cells from *Arabidopsis* leaves after immunogold labeling of cysteine**. Different amounts of gold particles can be observed between the *Arabidopsis* wild-type Col-0 **(A)**, the *pad 2-1* mutant **(B)**, the complemented line *OE3* **(C)**, and Col-0 after the treatment with 2mM buthionine sulfoximine (BSO) for 48 h **(D)**. Note that cells of the *OE3* line **(C)** show similar amounts of gold particles bound to cysteine in all organelles, whereas the *pad 2-1* mutant **(C)** and wild type plants treated with BSO **(D)** contain higher amounts of gold particles bound to cysteine in all cell compartments when compared to Col-0 **(A)**. C, chloroplasts; CW, cell walls; IS, intercellular spaces; M, mitochondria; N, nuclei; Px, peroxisomes; St, starch; V, vacuoles. Sections were post stained with uranyl acetate for 15 s. Bars: 1 μm.

**Figure 2:**
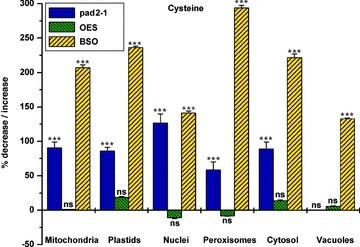
Quantitative analysis of cysteine specific labeling in the glutathione deficient mutant *pad2-1,* the complemented line *OE3* and after the treatment of wild type plants with buthionine sulfoximine (BSO). Graph shows means with standard errors and documents changes in the density of gold particles bound to cysteine in the respective *Arabidopsis* leaf cells when compared to control wildtype plants. Significant differences were calculated using the Mann-Whitney *U*-test; (***) indicates significance at the 0.001 level of confidence. *P* > 0.05 was considered as not significant (ns); *n* > 20 for peroxisomes and vacuoles and *n* > 60 for all other cell structures.

In wild type plants (accession Col-0) cysteine contents were found to be highest in mitochondria, peroxisomes and the cytosol. Significantly lower levels in this accession were found in plastids, nuclei and vacuoles (Table 1), whereas no cysteine was detected in cell walls and intercellular spaces (Figure 1). Significantly increased amounts of cysteine were found in most cell compartments of the *pad2-1* mutant, whereas similar levels of cysteine were found in all cell compartments of the complemented line OE3 when compared with wild type. The strongest increase of cysteine in the *pad2-1* mutant when compared with the wild type was found in nuclei (127%), followed by mitochondria and the cytosol (both 90%), plastids (86%) and peroxisomes (60%). Unchanged levels were found in vacuoles (Figures 1, 2). The treatment of wild type plants with BSO induced a strong increase in cysteine contents in peroxisomes (294%), plastids (236%), cytosol (222%), mitochondria (207%), nuclei (141%) and vacuoles when compared with control wild type plants (133%; Figure 2).

**Table 1:** Quantitative analysis of glutathione precursor specific labeling in *Arabidopsis* wild type plants

	Cysteine	Glutamate	γ-EC	Glycine
Mitochondria	8.3 ± 0.8^a^	37.4 ± 6^c^	70.4 ± 12^a^	12.8 ± 1^b^
Plastids	4.3 ± 0.3^b^	67 ± 2^b^	63 ± 4^a^	8.9 ± 0.3^cd^
Nuclei	4.5 ± 0.4^b^	83.7 ± 3^a^	71 ± 8^a^	10.3 ± 1^bc^
Peroxisomes	6.3 ±0.3^a^	65.2 ± 5^b^	61 ± 7^a^	12.2 ± 1^b^
Cytosol	7.2 ± 0.6^a^	72.6 ± 4^ab^	70 ± 7^a^	17 ± 1^a^
Vacuoles	3.4 ± 0.2^b^	n.d.	18 ± 0.7^b^	7.1 ± 0.4^d^

Data are means with standard errors and document changes in the density of gold particles per μm^2^ bound to cysteine, glutamate, γ-EC and glycine in the respective *Arabidopsis* leaf cells. Significant differences between the samples are indicated by different lowercase letters; samples that are significantly different from each other have no letter in common. *P* < 0.05 was regarded significant analyzed by the Kruskal-Wallis test, followed by post hoc comparison according to Conover. *n* > 20 for peroxisomes and vacuoles and *n* > 60 for all other cell structures. n.d., not detected.

### Glutamate

**Figure 3:**
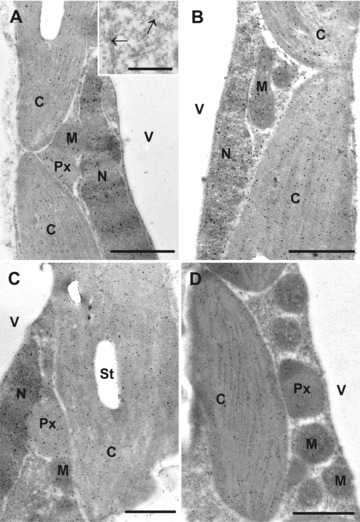
Transmission electron micrographs of mesophyll cells from *Arabidopsis* leaves after immunogold labeling of glutamate. Different amounts of gold particles can be observed between the *Arabidopsis* wild-type Col-0 **(A)**, the *pad 2-1* mutant **(B)**, the complemented line *OE3* **(C)**, and Col-0 after the treatment with 2mM buthionine sulfoximine (BSO) for 48 h **(D)**. Note that cells of the *pad 2-1* mutant **(B)** show slightly elevated levels of glutamate in most cell compartments, whereas the complemented line **(C)** and Col-0 plants treated with 2mM BSO for 48 h show increased levels only in mitochondria (M) and in chloroplasts and the cytosol, respectively, when compared to Col-0 **(A)**. Inset in A shows gold particles bound to glutamate along the membranes of the endoplasmic reticulum (arrows). C, chloroplasts; CW, cell walls; IS, intercellular spaces; M, mitochondria; N, nuclei; Px, peroxisomes; St, starch; V, vacuoles. Sections were post stained with uranyl acetate for 15 s. Bars: 1 μm and 0.5 μm in inset.

**Figure 4:**
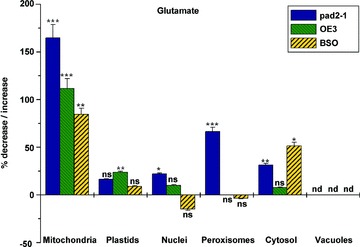
Quantitative analysis of glutamate specific labeling in the glutathione deficient mutant *pad2-1,* the complemented line *OE3* and after the treatment of wildtype plants with buthionine sulfoximine (BSO). Graph shows means with standard errors and documents changes in the density of gold particles bound to glutamate in the respective *Arabidopsis* leaf cells when compared to control wildtype plants. Significant differences were calculated using the Mann-Whitney *U*-test; (*), (**), and (***), respectively, indicate significance at the 0.05, 0.01, 0.001 level of confidence. *P* > 0.05 was considered as not significant (ns); *n* > 20 for peroxisomes and vacuoles and *n* > 60 for all other cell structures. nd, not detected.

In wild type plants (accession Col-0) glutamate-specific labeling was greatest in the nuclei and cytosol and lowest in mitochondria. Intermediate labeling in this accession was found in plastids and peroxisomes (Table 1), whereas no glutamate was detected in vacuoles, cell walls, or intercellular spaces (Figure 3). Glutamate could also be detected along the membranes of the endoplasmic reticulum (inset in Figure 3A). Glutamate-specific labeling significantly increased in most cell compartments of the *pad2-1* mutant when compared to wild type plants. In the *pad2-1* mutant, the strongest increase in glutamate contents was observed in mitochondria (165%), followed by peroxisomes (66%), the cytosol (31%), and nuclei (22%). No significant change in glutamate specific labeling was observed in plastids in the *pad2-1* mutant when compared to wild type. In the complemented line, OE3 glutamate was significantly increased in mitochondria (112%) and plastids (21%) when compared to wild type. No significant change was found in nuclei, peroxisomes or the cytosol in the complemented line when compared to the wild type (Figures 3,4). BSO-treatment of wild type plants caused a significant increase in glutamate contents in comparison to control wild type plants in mitochondria (84%) and the cytosol (52%) whereas glutamate contents in all other cell compartments remained unaffected (Figure 4).

### γ-EC

**Figure 5:**
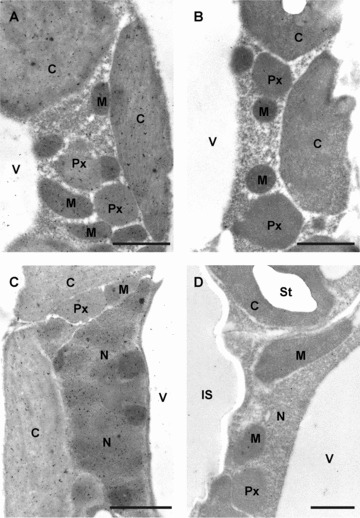
Transmission electron micrographs of mesophyll cells from *Arabidopsis* leaves after immunogold labeling of γ-glutamylcysteine (γ-EC). Different amounts of gold particles can be observed between the *Arabidopsis* wild-type Col-0 (A), the *pad 2-1* mutant (B), the complemented line *OE3* (C), and Col-0 after the treatment with 2mM buthionine sulfoximine (BSO) for 48 h (D). Note that cells of the *OE3* line (C) show similar amounts of gold particles bound to γ-EC in all organelles, whereas the *pad 2-1* mutant (B) and wild type plants treated with BSO (D) contain much lower amounts of gold particles bound to γ-EC in all cell compartments when compared to Col-0 (A). C, chloroplasts; CW, cell walls; IS, intercellular spaces; M, mitochondria; N, nuclei; Px, peroxisomes; St, starch; V, vacuoles. Sections were post stained with uranyl acetate for 15 s. Bars: 1 μm.

**Figure 6:**
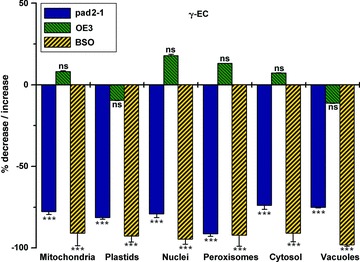
Quantitative analysis of γ-glutamylcysteine (γ-EC)specific labeling in the glutathione deficient mutant *pad2-1,* the complemented line *OE3* and after the treatment of wild type plants with buthionine sulfoximine (BSO). Graph shows means with standard errors and documents changes in the density of gold particles bound to γ-EC in the respective *Arabidopsis* leaf cells when compared to control wildtype plants. Significant differences were calculated using the Mann-Whitney *U*-test; (***) indicates significance at the 0.001 level of confidence. *P* > 0.05 was considered as not significant (ns); *n* > 20 for peroxisomes and vacuoles and *n* > 60 for all other cell structures.

In wild type plants (Col-0), the greatest level of labeling density of γ-EC was detected in nuclei, which did not significantly differ from γ-EC-specific labeling in mitochondria, plastids, nuclei, peroxisomes and the cytosol (Table 1). Lowest levels of γ-EC were detected in vacuoles and γ-EC labeling was absent in cell walls and intercellular spaces in wild type plants (Figure 5). Compartment-specific labeling of γ-EC was strongly decreased in all cell compartments of the *pad2-1* mutant and remained unchanged in all cell compartments of the complemented line OE3 when labeling density was compared to the wild type. In the *pad2-1* mutant, the strongest decrease of γ-EC was observed in peroxisomes (–88%) followed by plastids (–82), nuclei (–79%), mitochondria (77), the cytosol (–74%) and vacuoles (–75%) when compared to the wild type (Figures 5,6). The treatment of wild type plants with BSO caused a strong decrease in γ-EC contents in all cell compartments between 91% and 98% in comparison to control wild type plants (Figure 6).

### Glycine

**Figure 7:**
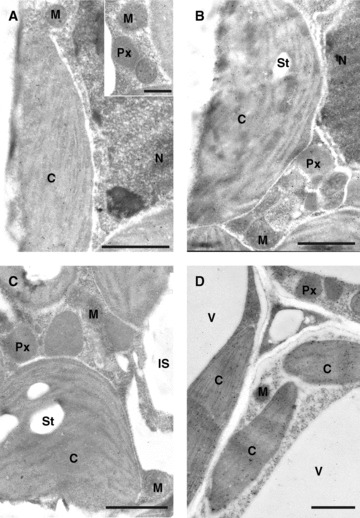
Transmission electron micrographs of mesophyll cells from *Arabidopsis* leaves after immunogold labeling of glycine. Different amounts of gold particles can be observed between the *Arabidopsis* wild-type Col-0 (A), the *pad 2-1* mutant (B), the complemented line *OE3* (C), and Col-0 after the treatment with 2 mM buthionine sulfoximine (BSO) for 48 h (D). Note that cells of the *OE3* line (C) show similar amounts of gold particles bound to glycine whereas the *pad 2-1* mutant (B) and wild type plants treated with BSO (D) contain higher amounts of gold particles bound to glycine in most cell compartments when compared to Col-0 (A). C, chloroplasts; CW, cell walls; IS, intercellular spaces; M, mitochondria; N, nuclei; Px, peroxisomes; St, starch; V, vacuoles. Sections were post stained with uranyl acetate for 15 s. Bars: 1 μm.

**Figure 8:**
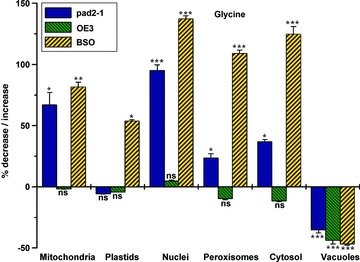
Quantitative analysis of glycine specific labeling in the glutathione deficient mutant *pad2-1,* the complemented line *OE3* and after the treatment of wildtype plants with buthionine sulfoximine (BSO). Graph shows means with standard errors and documents changes in the density of gold particles bound to glycine in the respective *Arabidopsis* leaf cells when compared to control wildtype plants. Significant differences were calculated using the Mann-Whitney *U*-test; (*), (**), and (***), respectively, indicate significance at the 0.05, 0.01, 0.001 level of confidence. *P* > 0.05 was considered as not significant (ns); *n* > 20 for peroxisomes and vacuoles and *n* > 60 for all other cell structures.

In wild type plants (Col-0), the greatest level of glycine-specific labeling was detected in the cytosol. Intermediate labeling in this accession was found in mitochondria, nuclei and peroxisomes, which contained significantly less glycine than the cytosol (Table 1). The lowest glycine-specific labeling intensity was detected in plastids and vacuoles (Figure 7). A strong increase in glycine-specific labeling was observed in most cell compartments of the *pad2-1* mutant when compared to the wild type, whereas in the complemented line OE3 unchanged levels were found in most cell compartments. In the *pad2-1* mutant, the strongest increase in glycine-specific labeling was detected in nuclei (95%), followed by mitochondria (67%), the cytosol (37%) and peroxisomes (24%) when compared to wild type plants. Whereas unchanged levels of glycine were found in plastids of the *pad2-1* mutant, a decrease was found in vacuoles (−44%) in comparison to wild type plants. In the complemented lines, vacuoles showed a decrease in glycine-specific labeling (−44%), whereas glycine contents in the other cell compartments remained unchanged when glycine labeling was compared to the wild type (Figures 7,8). BSO-treatment of wild type plants caused a massive increase in glycine contents in all cell compartments when labeling levels were compared to control wild type plants. Glycine was increased in nuclei (137%), the cytosol (125%), peroxisomes (109%), mitochondria (82%) and plastids (54%). A decrease was observed in vacuoles (–47%; Figure 8).

### Glutathione

The subcellular distribution of glutathione in *Arabidopsis* accession Col-0, the glutathione-deficient *Arabidopsis* mutant *pad2-1* and the complemented line OE3 has been described previously in detail (Zechmann et al. 2008b). Nevertheless, parts of the data described previously in addition to data obtained during BSO treatment (2 mM for 48 h) have been included in this manuscript in order to allow a better correlation of changes in glutathione precursor contents with glutathione levels under these conditions. These results show that glutathione contents in leaves of the *pad2-1* mutant were decreased when compared with wild type plants by about 92% in nuclei, 91% in peroxisomes, 86% in the cytosol, and 84% in plastids (Supplemental Figure 2). No significant change was found in mitochondria. In leaves of the complemented line OE3, gold particle density increased between 153% in peroxisomes, 125% in plastids, 78% in the cytosol, 52% in nuclei, and 43% in mitochondria in comparison to wild type. BSO-treatment of wild type plants significantly decreased glutathione contents in nuclei (–100%), peroxisomes (–100%), mitochondria (–96%), the cytosol (–92%) and in plastids (–76%) when compared with control wild type plants (Supplemental Figure 1).

## Discussion

The subcellular distribution of glutathione precursors was studied by high resolution immuno electron microscopy in *Arabidopsis* plants. These studies revealed that all glutathione precursors were absent in the apoplast, but could be detected in different concentrations in the cytoplasm and nuclei of the cells (with the exception of glutamate in vacuoles). These results are similar to what has been found previously in pumpkin plants (Zechmann et al. 2006a, 2007b; Zechmann and Müller 2008) and extend the current knowledge of glutathione synthesis and degradation in plants.

The occurrence of glycine, cysteine and γ-EC, but not glutamate, in vacuoles of wild type plants indicates that glutathione could be degraded in two possible pathways within the vacuole and/or tonoplast. The first possible pathway for glutathione degradation within vacuoles could be triggered by GGT, which transfers glutamate to other dipeptides and leaves the dipeptide Cys-Gly, which could then be metabolized by a dipeptidase to its components (Foyer et al. 2001; Storozhenko et al. 2002; Shaw et al. 2005; Ohkama-Ohtsu et al. 2007a, 2007b). One isoform of GGT (GGT4 which was originally called GGT3 by Ohkama-Ohtsu et al. 2007a, 2007b but is refereed to GGT4 in the latest literature) has been detected within vacuoles (Ohkama-Ohtsu et al. 2007b; Ohkama-Ohtsu et al. 2008; Destro et al. 2011; Noctor et al. 2011) and could be responsible for the degradation of glutathione and glutathione-conjugates in this organelle.

The second possible pathway of glutathione degradation in vacuoles would involve the cleavage of glycine by carboxypeptidase (Steinkamp and Rennenberg 1985), which has been detected in vacuoles of tomato (Wolf et al. 1996) but still has yet to be established for *Arabidopsis thaliana*. Both of these reactions would leave cysteine, glycine and γ-EC in vacuoles. As glutamate would be transferred to other dipeptides it could not be detected with the method used in this study.

Glutathione and its precursors were absent in the apoplast of wild type plants, which indicates that glutathione in the apoplast of *Arabidopsis thaliana* is degraded very rapidly by GGT and that the degradation products are transported back into the cytosol. Two isoforms of the enzyme (GGT1 and GGT2) responsible for glutathione degradation in the apoplast have been found to be associated or bound to the plasmalemma in *Arabidopsis thaliana* (Ohkama-Ohtsu et al. 2007a; Destro et al. 2011). While GGT2 was detected only in young siliques (Ohkama-Ohtsu et al. 2007a) and roots (Destro et al. 2011), GGT1 activity was also detected in leaves. In light of these results, we propose that glutathione degradation in *A. thaliana* leaves is performed by GGT1 at the plasmalemma and that the resulting products (Cys-Gly and glutamate) are either further metabolized by dipeptidases or rapidly transported back into the cytoplasm. Within individual cells, glutathione precursors were found in all cell compartments in different concentrations.

Glutathione synthesis strongly depends on the availability of its precursors in glutathione synthesizing organelles such as plastids and the cytosol (Noctor et al. 2011) where glutathione precursor levels were present in intermediate concentrations in the accession Col-0. Very little is known about their subcellular distribution during impaired glutathione synthesis. In the present study we were able to demonstrate that the inhibition of the first enzyme of glutathione synthesis, either through BSO-treatment or in the GSH1 single point mutant *pad2-1*, led to the accumulation of cysteine, glycine and glutamate in most cell compartments including plastids and the cytosol when labeling intensity was compared to wild type plants. A decrease of γ-EC and glutathione was observed in all cell compartments. Expectantly, plastids, which are considered to be the main production center for γ-EC in *Arabidopsis* (Wachter et al. 2005; Noctor et al. 2011), showed a strong decrease (over 80%) of this glutathione precursor in the glutathione deficient *pad2-1* mutant in comparison to wild type plants of accession Col-0. This observation could be correlated with a strong decrease also in all other cell compartments. A similar situation was observed when plants were treated with BSO, which specifically inhibits GSH1. Thus, we can conclude that low levels of γ-EC, induced by the inhibition of GSH1 in the *pad2-1* mutant and during BSO-treatment, limit glutathione synthesis, thus leading to a strong decrease in total glutathione contents. The complemented line OE3, with restored glutathione synthesis and glutathione contents similar to the wild type (Parisy et al. 2007; Zechmann et al. 2008a), showed similar or slightly increased labeling density of cysteine, glutamate, γ-EC, and glycine in all cell compartments in comparison to wild type plants. These results demonstrate that the complementation of the *pad2-1* mutant with GSH1 cDNA from the wild type restored not only glutathione contents, but also levels of glutathione precursors in most cell compartments. Additionally, we can conclude from these studies that the antibody methods used in this study specifically detect changes in the desired glutathione precursors as glutathione precursor contents could be detected in concentrations that correlated well with the expected concentrations due to the modulation experiments.

Summing up, we can conclude that low glutathione contents induced by the inhibition of GSH1 in the *pad2-1* mutant and during BSO-treatment are caused by low γ-EC contents in most cell compartments including glutathione producing organelles such as chloroplasts and the cytosol. The distribution of glutathione precursors in *A. thaliana* accession Col-0 is limited to the protoplast, which indicates an effective degradation of glutathione by GGT in the apoplast and that the resulting amino acids are rapidly transported back into the cytosol.

## Material and Methods

### Plant material

The experiments in this study were performed with *Arabidopsis thaliana* (L.) Heynh. ecotype Columbia (Col-0), the *pad 2-1* (phytoalexin deficient) mutant and one transgenic line (OE3) overexpressing GSH1. The *pad 2-1* mutant is characterized as having a single point mutation in gene At4g23100 that encodes GSH1, which catalyses the first step of glutathione synthesis (Parisy et al. 2007). The single point mutation in *pad2-1* is characterized by a single G to A nucleotide transition at position 1 697 from the start codon of the gene At4g23100. This mutation leads to the replacement of a serine by an asparagine residue at position 298 in the 522 amino-acid protein (Parisy et al. 2007). The transgenic line OE3, which is equivalent to line 3 in Parisy et al 2007, was genetically complemented by using *cDNA* from GSH1 of *Arabidopsis* and insertion into the *pad2-1* mutant as described in detail by Parisy et al. 2007. While glutathione contents are strongly decreased in the *pad2-1* mutant, they are restored in the complemented line and reach or exceed values of the wild type (Parisy et al. 2007; Zechmann et al. 2008b).

All plants were cultivated in growth chambers under defined conditions. After stratification for 4 days at 4 °C seeds of the *Arabidopsis* mutant *pad2-1* were grown on soil in growth chambers with 14:10 h light: dark (L:D) photoperiod. Day and night temperatures were 22 °C and 18 °C, respectively, the relative humidity was 60% and the plants were kept at 100% relative soil water content. Light intensity varied between 110 and 140 μmol/m^2^ per s. Plants were kept in pots with soil and were watered adequately. Harvesting of *Arabidopsis* plants was performed 4 weeks after stratification. Therefore, the youngest fully developed rosette leaves were harvested 2 h after the onset of the light period. Leaves at this stage were approximately 2 cm in length and 0.7 cm in width. Immunocytochemical methods were used to determine the subcellular distribution of cysteine, glutamate, glycine, γ-EC and glutathione in leaves of *Arabidopsis*.

### BSO treatment

Four-week-old *Arabidopsis* plants raised in soil were treated with 2 mM L-buthionine [S,R] sulfoximine (BSO; Sigma-Aldrich, St. Louis, MO, USA) dissolved in distilled water (pH 7.2) for 48 h. A control group was treated with the same solution without BSO. The BSO-solution was exchanged every 12 h. Treatment was carried out in plastic dishes (100 mL) covered with a nylon mesh by immersing the roots into the solutions. For treatment, seedlings were carefully removed from the pots filled with soil and the roots were gently rinsed with tap water (pH 7.2) until the soil was washed off. The roots were then immersed into the solution, whereas the stems and leaves were held by the nylon mesh above the solution.

### Sample preparation for transmission electron microscopy and immunogold labeling

Preparation of samples for transmission electron microscopy (TEM) and immunogold labeling of glutathione was done with ultrathin sections on nickel grids as described in Zechmann et al. (2006a, 2006b, 2008b). Small samples of the youngest fully developed leaves (about 1.5 mm^2^) from at least three different plants were fixed in 2.5% paraformaldehyde/0.5% glutardialdehyde in 0.06M phosphate buffer (pH 7.2) for 90 min at room temperature (RT). Samples were then rinsed in buffer and dehydrated in increasing concentrations of acetone (50%, 70%, and 90%) at RT for 20 min at each step. Subsequently, specimens were gradually infiltrated with increasing concentrations of LR-White resin (30%, 60% and 100%; London Resin Company Ltd., Berkshire, UK) mixed with acetone (90%) and finally embedded in LR-White resin and polymerized at 50 °C for 48 h in small plastic containers.

Ultrathin sections (80 nm) of the samples were blocked with 2% bovine serum albumine (BSA, Sigma-Aldrich) in phosphate buffered saline (PBS, pH 7.2), then treated with the primary antibodies (anti-glutathione rabbit polyclonal IgG, anti-cysteine rabbit polyclonal IgG, anti-glutamate rabbit polyclonal IgG, anti-glycine rabbit polyclonal IgG, Millipore Corp., (Billerica, MA, USA); anti-γ-glutamylcysteine rabbit polyclonal IgG produced by Agrisera, Vännäs, Sweden) diluted 1:50 (anti-glycine and anti-γ-glutamylcysteine) and 1:300 (anti-cysteine and anti-glutamate) in PBS containing 1% goat serum for 2 h at RT. After a short rinse in PBS, samples were incubated with a 10 nm gold-conjugated secondary antibody (goat anti-rabbit IgG, British BioCell International, Cardiff, UK) diluted 1:50 (anti-glutathione, anti-cysteine and anti- γ-glutamylcysteine) and 1:100 (anti-glutamate and anti-glycine) in PBS for 90 min at RT. After a short wash in PBS, and distilled water labeled grids were either immediately observed under a Philips CM10 transmission electron microscope or post-stained with uranyl-acetate (15 s).

The specificity of the immunogold labeling procedures was tested by several negative controls. Negative controls were treated either with: (i) pre-immune serum instead of the primary antibody; (ii) gold conjugated secondary antibody (goat anti-rabbit IgG) without the primary antibody; (iii) non-specific secondary antibody (goat anti-mouse IgG); and (iv) primary antibodies pre-absorbed with an excess of either glutathione, cysteine, glutamate, glycine or γ-EC for 2 h at RT prior to labeling of the sections. For the latter solutions containing either 10 mM of glutathione, cysteine, glutamic acid, glycine or γ-EC were incubated with 0.5% glutardialdehyde for one hour. The excess of glutardialdehyde was then saturated by incubation for 30 min in a solution of 1% (w/v) BSA. The resulting solutions were then used to saturate the respective antibodies for 2 h prior to its use.

Negative controls for cysteine (Supplemental Figure 2), glutamate (Supplemental Figure 3), γ-EC (Supplemental Figure 4) and glycine (Supplemental Figure 5) revealed that no labeling occurred on the section when they were treated with (i) pre-immune serum instead of the primary antibody (image a in Supplemental Figures 2–5); (ii) gold conjugated secondary antibody without the primary antibody (image b in Supplemental Figures 2–5); (iii) non-specific secondary antibody (image c in Supplemental Figures 2–5); and (iv) primary antibodies pre-absorbed with an excess of either cysteine, glutamic acid, glycine or γ-EC for 2 h at RT prior to labeling of the sections (image d in Supplemental Figures 2–5). Negative controls for the glutathione antibody have been published previously (Zechmann et al. 2005, 2008b; Zechmann and Müller 2010).

In order to test if the antibodies against cysteine and glycine also bind to the dipeptide cys-gly the antibodies were incubated with a 10 mM cys-gly solution (Sigma-Aldrich) for 2 h. Subsequently the antibodies were then applied on sections of the wild type. Labeling results revealed that the pre-incubation of the cysteine, and glycine antibodies with cys-gly (Supplemental Figure 6) did not induce changes in cysteine and glycine labeling as gold particle density was similar to what was observed in wild type without the pre-incubation of the antibodies with cys-gly (Figures 1A,7A). Thus, these results demonstrate that the antibodies against cysteine and glycine do not bind to the dipeptide cys-gly, otherwise labeling would have been strongly decreased after the incubation as cys-gly would have bound and subsequently blocked the binding sites of the antibodies.

Micrographs of randomly photographed immunogold-labeled sections were digitized and gold particles were counted automatically using the software package Cell D with the particle analysis tool (Olympus, Life and Material Science Europa GmbH, Hamburg, Germany) in manually identified cell structures. For statistical evaluation, at least four different samples were examined for each treatment or mutant. From these samples a minimum of 20 (peroxisomes and vacuoles) to 60 (other cell structures) sectioned cell structures of at least 15 different cells were analyzed for gold particle density per treatment. The obtained data were statistically evaluated using Statistica (Stat-Soft Europe, Hamburg, Germany). The obtained data were presented as the number of gold particles per μm^2^. For statistical analyses either the Mann-Whitney U-test or the non-parametric Kruskal-Wallis test followed by a post-hoc comparison according to Conover was used. *P* < 0.05 was considered as significant (Bortz et al. 2008).

(Co-Editor: Katie Dehesh)

## Supporting Information

Supplemental Figure 1 Quantitative analysis ofglutathione specific labeling in the glutathione deficient mutant*pad2-1,* the complemented line *OE3* and after thetreatment of wild type plants with BSO. Graph shows means withstandard errors and documents changes in the density of goldparticles bound to glutathione in the respective *Arabidopsis*leaf cells when compared to control wildtype plants. Significantdifferences were calculated using the Mann-Whitney *U*-test;(*), (**), and (***), respectively, indicate significance at the0.05, 0.01, 0.001 level of confidence. *P* > 0.05 wasconsidered as not significant (ns); *n* > 20 forperoxisomes and vacuoles and *n* > 60 for all other cell structures.

Supplemental Figure 2 Transmission electron micrographsof mesophyll cells from *Arabidopsis* leaves treated asnegative control for cysteine labeling. Gold particles were absentwhen cells were treated with pre-imune serum instead of the primaryantibody (a), after the omission of the primary antibody (b), withan unspecific secondary antibody (c) and the cysteine antibodypre-absorpt with an excess of cysteine prior to its application(d). C, chloroplasts; CW, cell walls; IS, intercellular spaces; M,mitochondria; N, nuclei; Px, peroxisomes; St, starch; V, vacuoles.Sections were post stained with uranyl acetate for 15 s. Bars: 1μm.

Supplemental Figure 3 Transmission electron micrographsof mesophyll cells from *Arabidopsis* leaves treated asnegative control for glutamate labeling. Gold particles were absentwhen cells were treated with pre-immune serum instead of theprimary antibody (a), after the omission of the primary antibody(b), with an unspecific secondary antibody (c) and the glutamateantibody pre-absorpt with an excess of glutamic acid prior to itsapplication (d). C, chloroplasts; CW, cell walls; IS, intercellularspaces; M, mitochondria; N, nuclei; Px, peroxisomes; St, starch; V,vacuoles. Sections were post stained with uranyl acetate for 15 s.Bars: 1 μm.

Supplemental Figure 4 Transmission electron micrographsof mesophyll cells from *Arabidopsis* leaves treated asnegative control for γ-EC labeling. Gold particles wereabsent when cells were treated with pre-imune serum instead of theprimary antibody (a), after the omission of the primary antibody(b), with an unspecific secondary antibody (c) and the γ-ECantibody pre-absorpt with an excess of γ-EC prior to itsapplication (d). C, chloroplasts; CW, cell walls; IS, intercellularspaces; M, mitochondria; N, nuclei; Px, peroxisomes; St, starch; V,vacuoles. Sections were post stained with uranyl acetate for 15 s.Bars: 1 μm.

Supplemental Figure 5 Transmission electron micrographsof mesophyll cells from *Arabidopsis* leaves treated asnegative control for glycine labeling. Gold particles were absentwhen cells were treated with pre-imune serum instead of the primaryantibody (a), after the omission of the primary antibody (b), withan unspecific secondary antibody (c) and the glycine antibodypre-absorpt with an excess of glycine prior to its application (d).C, chloroplasts; CW, cell walls; IS, intercellular spaces; M,mitochondria; N, nuclei; Px, peroxisomes; St, starch; V, vacuoles.Sections were post stained with uranyl acetate for 15 second. Bars:1μm.

Supplemental Figure 6 Transmission electron micrographsof mesophyll cells from *Arabidopsis* leaves treated withantibodies against cysteine (a) and glycine (b) after incubation ofthe antibodies with a 10 mM cys-gly solution for 2 h prior to thelabeling experiment. Gold particle density was found to be similarin the different cell compartments when compared to the sections ofthe wildtype which were treated with antibodies against cysteine(Figure 1A) and glycine (Figure 7A) without pre-incubation ofcys-gly. C, chloroplasts; CW, cell walls; IS, intercellular spaces;M, mitochondria; N, nuclei; Px, peroxisomes; St, starch; V,vacuoles. Sections were post stained with uranyl acetate for 15 s.Bars: 1 μm and 0.5 μm in inset.
