# Supplementary figures and images for: Subcellular Distribution of Glutathione Precursors in Arabidopsis thaliana
Source: J Integr Plant Biol. 2011 Dec 13;53(12):930–41. doi: 10.1111/j.1744-7909.2011.01085.x (PMC3588602; doi:10.1111/j.1744-7909.2011.01085.x)

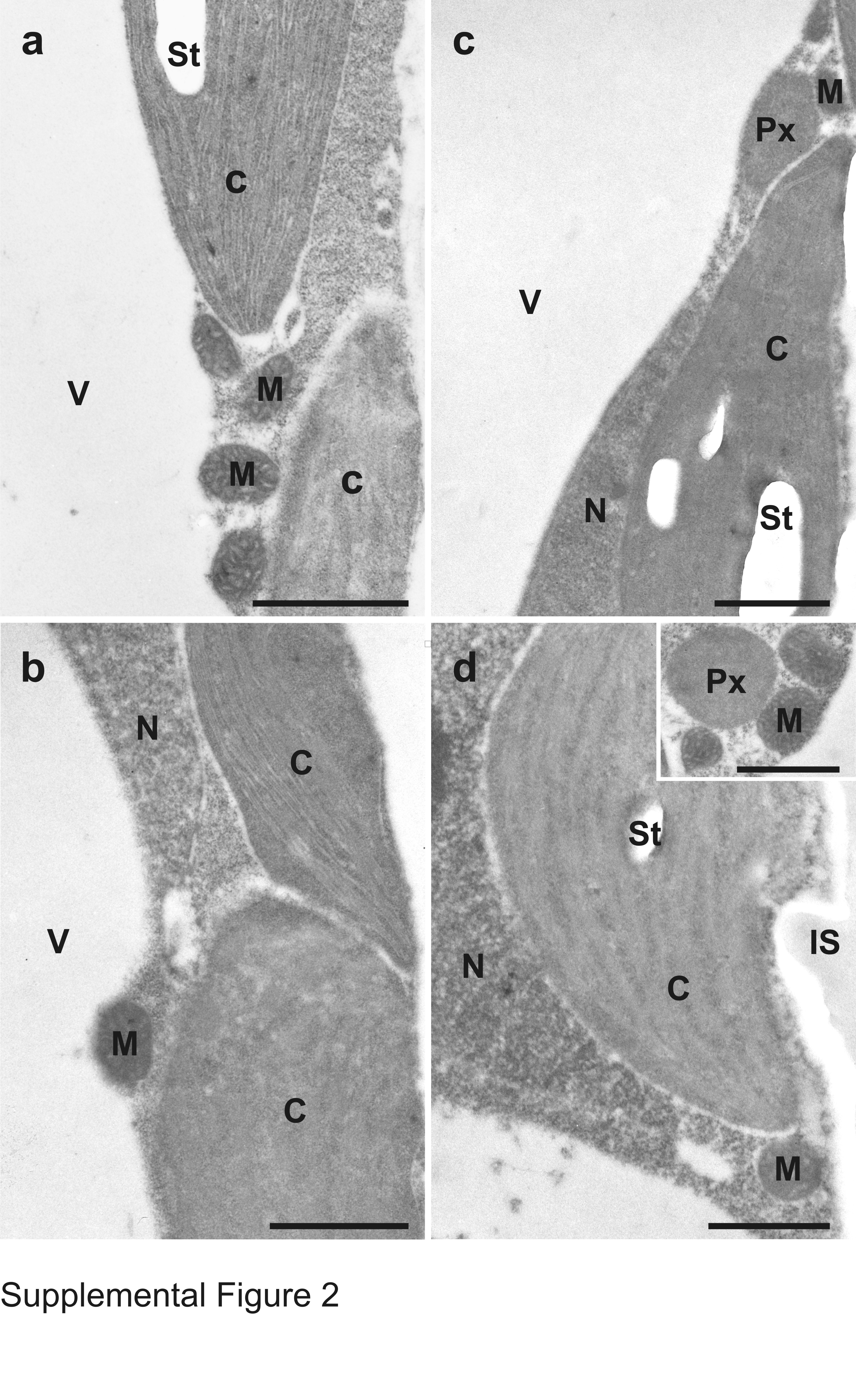

Supplement: Supplemental Figure 2 — Transmission electron micrographsof mesophyll cells from Arabidopsis leaves treated asnegative control for cysteine labeling. Gold particles were absentwhen cells were treated with pre-imune serum instead of the primaryantibody (a), after the omission of the primary antibody (b), withan unspecific secondary antibody (c) and the cysteine antibodypre-absorpt with an excess of cysteine prior to its application(d). C, chloroplasts; CW, cell walls; IS, intercellular spaces; M,mitochondria; N, nuclei; Px, peroxisomes; St, starch; V, vacuoles.Sections were post stained with uranyl acetate for 15 s. Bars: 1μm. [file jipb0053-0930-sd2.jpg]

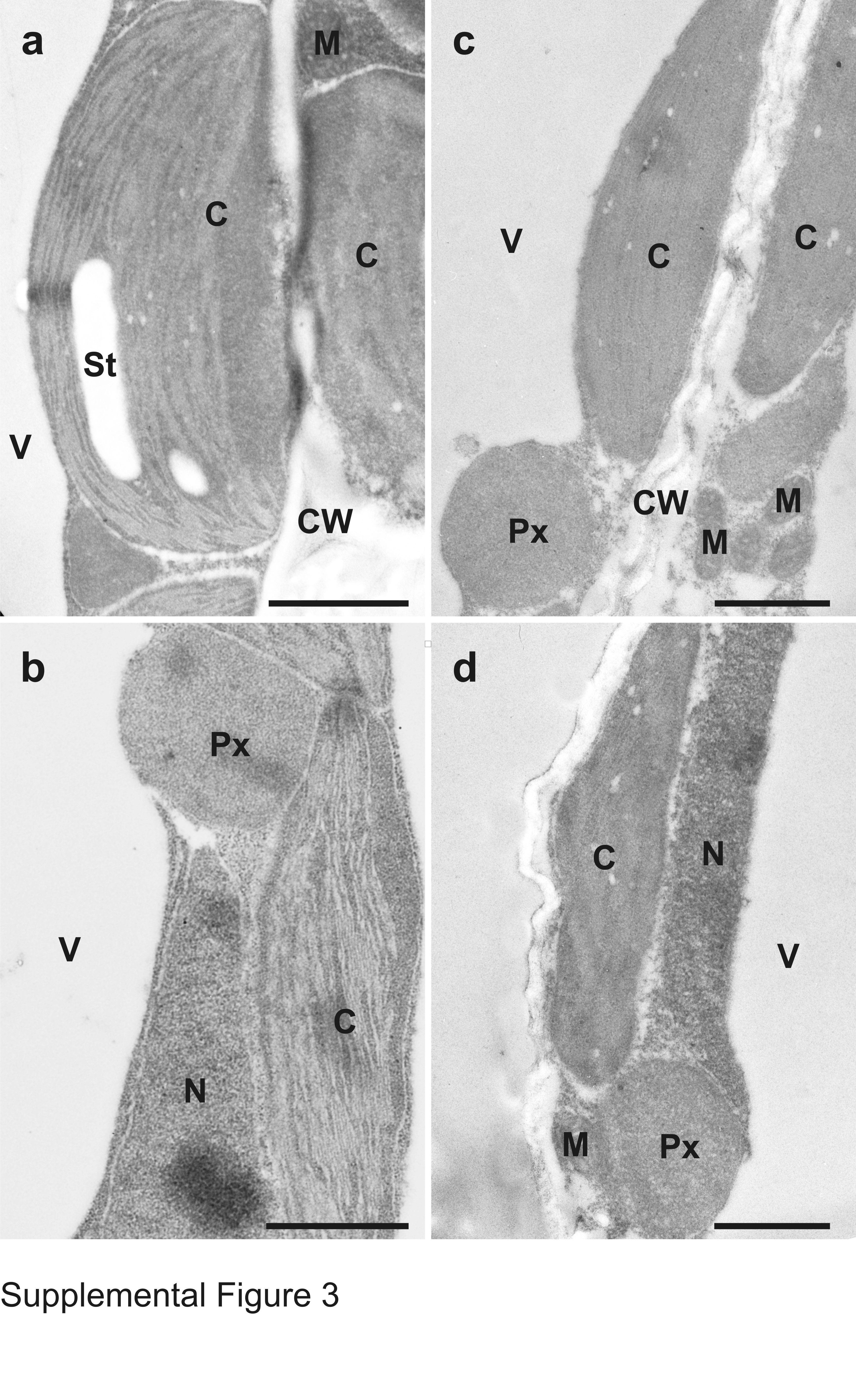

Supplement: Supplemental Figure 3 — Transmission electron micrographsof mesophyll cells from Arabidopsis leaves treated asnegative control for glutamate labeling. Gold particles were absentwhen cells were treated with pre-immune serum instead of theprimary antibody (a), after the omission of the primary antibody(b), with an unspecific secondary antibody (c) and the glutamateantibody pre-absorpt with an excess of glutamic acid prior to itsapplication (d). C, chloroplasts; CW, cell walls; IS, intercellularspaces; M, mitochondria; N, nuclei; Px, peroxisomes; St, starch; V,vacuoles. Sections were post stained with uranyl acetate for 15 s.Bars: 1 μm. [file jipb0053-0930-sd3.jpg]

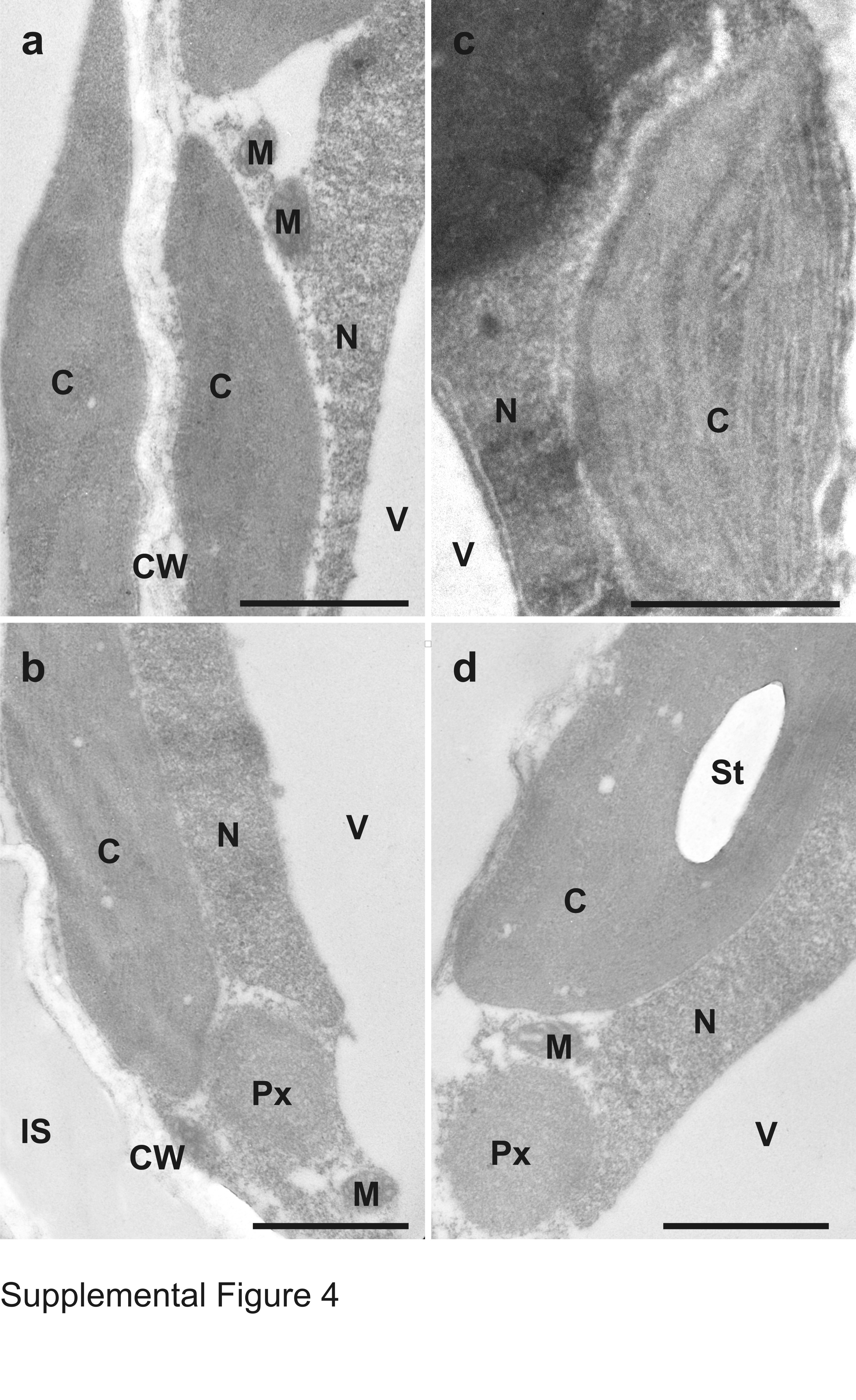

Supplement: Supplemental Figure 4 — Transmission electron micrographsof mesophyll cells from Arabidopsis leaves treated asnegative control for γ-EC labeling. Gold particles wereabsent when cells were treated with pre-imune serum instead of theprimary antibody (a), after the omission of the primary antibody(b), with an unspecific secondary antibody (c) and the γ-ECantibody pre-absorpt with an excess of γ-EC prior to itsapplication (d). C, chloroplasts; CW, cell walls; IS, intercellularspaces; M, mitochondria; N, nuclei; Px, peroxisomes; St, starch; V,vacuoles. Sections were post stained with uranyl acetate for 15 s.Bars: 1 μm. [file jipb0053-0930-sd4.jpg]

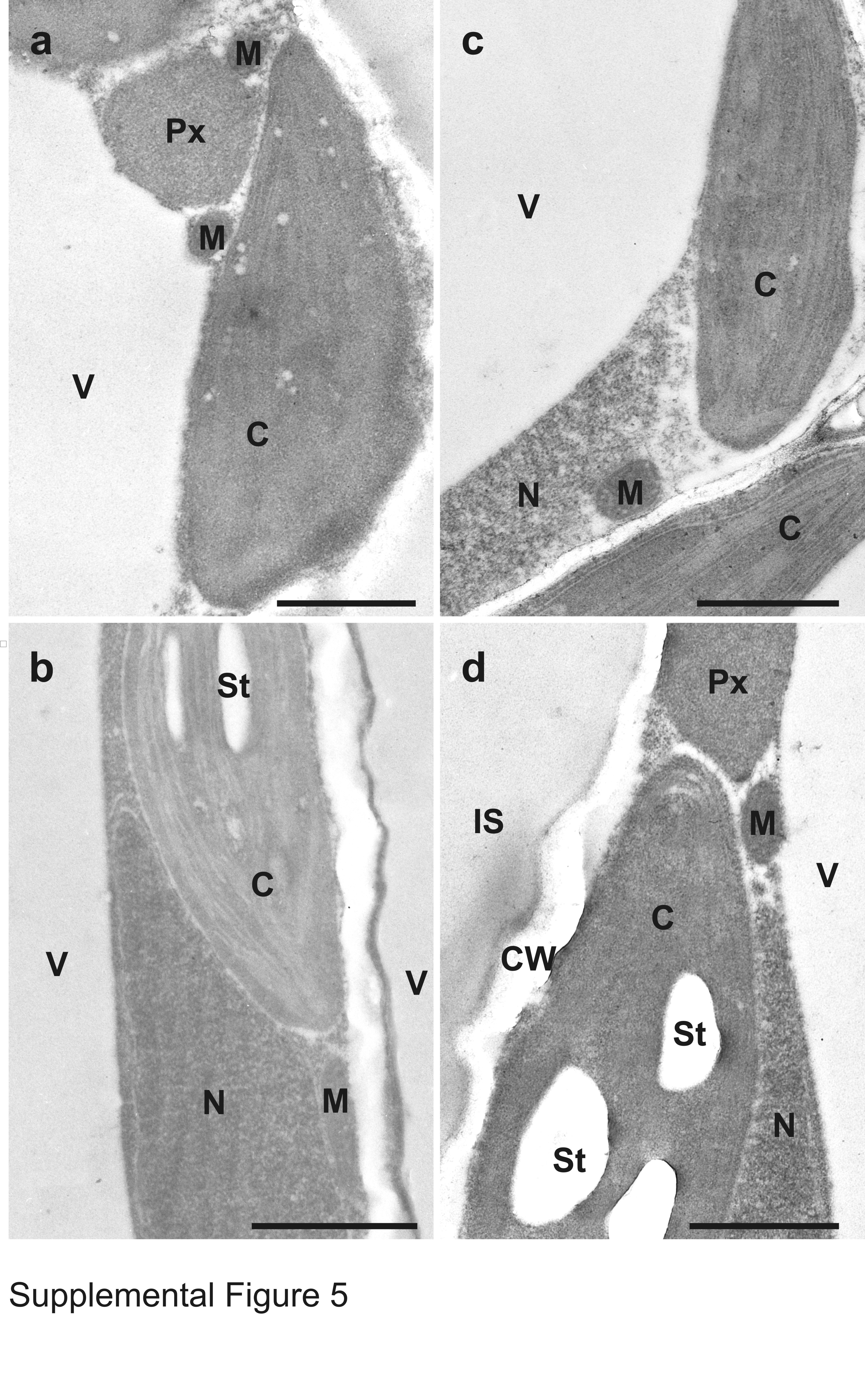

Supplement: Supplemental Figure 5 — Transmission electron micrographsof mesophyll cells from Arabidopsis leaves treated asnegative control for glycine labeling. Gold particles were absentwhen cells were treated with pre-imune serum instead of the primaryantibody (a), after the omission of the primary antibody (b), withan unspecific secondary antibody (c) and the glycine antibodypre-absorpt with an excess of glycine prior to its application (d).C, chloroplasts; CW, cell walls; IS, intercellular spaces; M,mitochondria; N, nuclei; Px, peroxisomes; St, starch; V, vacuoles.Sections were post stained with uranyl acetate for 15 second. Bars:1μm. [file jipb0053-0930-sd5.jpg]

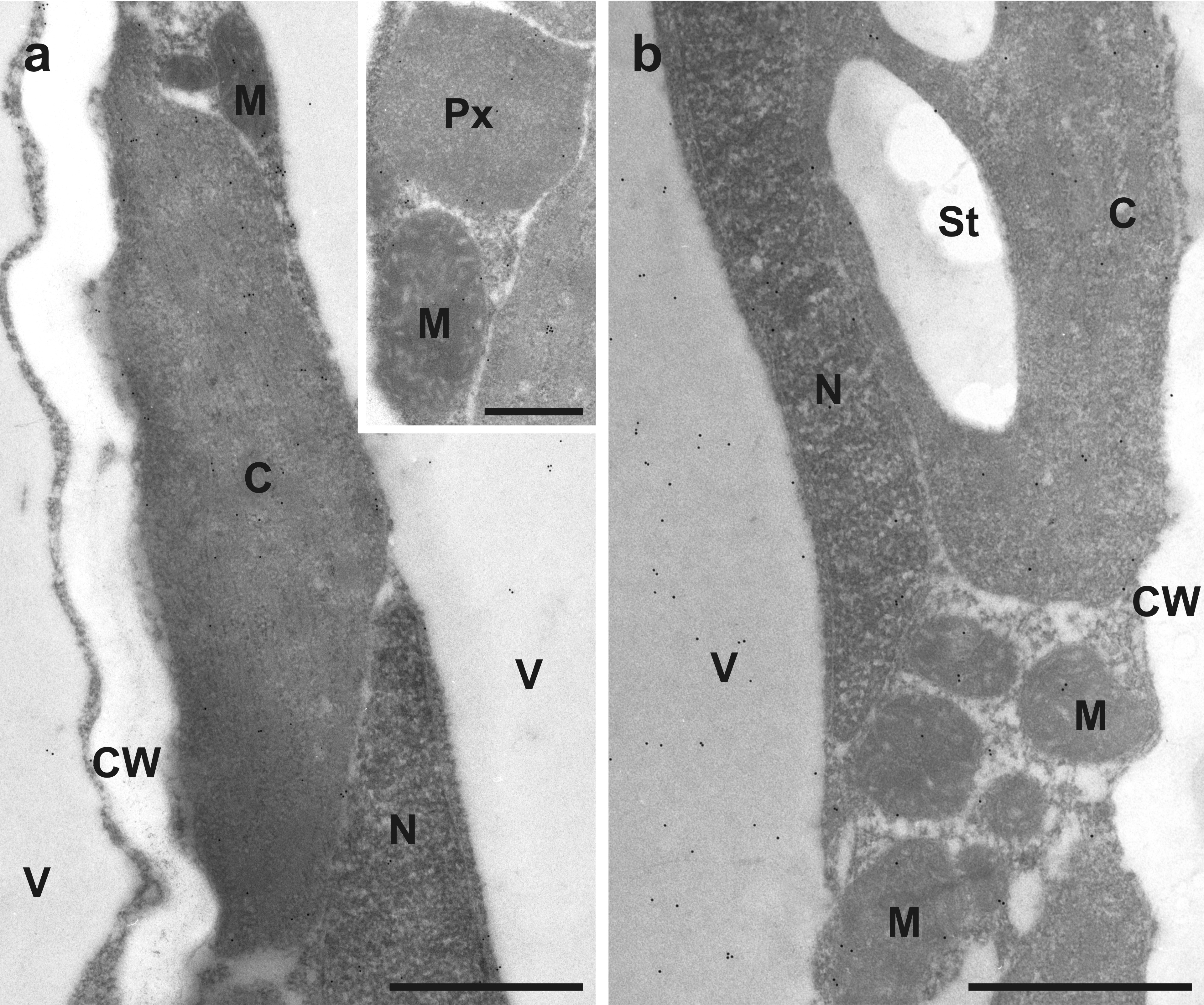

Supplement: Supplemental Figure 6 — Transmission electron micrographsof mesophyll cells from Arabidopsis leaves treated withantibodies against cysteine (a) and glycine (b) after incubation ofthe antibodies with a 10 mM cys-gly solution for 2 h prior to thelabeling experiment. Gold particle density was found to be similarin the different cell compartments when compared to the sections ofthe wildtype which were treated with antibodies against cysteine(Figure 1A) and glycine (Figure 7A) without pre-incubation ofcys-gly. C, chloroplasts; CW, cell walls; IS, intercellular spaces;M, mitochondria; N, nuclei; Px, peroxisomes; St, starch; V,vacuoles. Sections were post stained with uranyl acetate for 15 s.Bars: 1 μm and 0.5 μm in inset. [file jipb0053-0930-sd6.jpg]
